# Soft-Centralized Spectrum Resource Management in UAV-Assisted MANETs from Aggregate Multi-Hop Information Efficiency

**DOI:** 10.3390/s26051446

**Published:** 2026-02-26

**Authors:** Tianyi Zhang, Yang Zheng

**Affiliations:** 1 The College of Communication Engineering, Xidian University, Xi’an 710071, China; 2 State Key Laboratory of Integrated Service Networks, Xi’an 710071, China

**Keywords:** UAV-assisted MANETs, spectrum resource management, performance evaluation, AMIE, soft-centralized

## Abstract

UAV-Assisted Mobile Ad Hoc Networks (UAMANETs) provide flexible communication support in dynamic and infrastructure-limited environments. This paper studies a representative UAMANET architecture in which a subset of UAVs forms stable task clusters with ground nodes while simultaneously acting as relays in an airborne backbone network. To characterize the network capacity under contention-based medium access and multi-hop routing, we introduce Aggregate Multi-hop Information Efficiency (AMIE), a capacity-oriented metric that jointly accounts for MAC-layer contention, multi-hop routing, and end-to-end transmission reliability. Based on an IEEE 802.11p access model, we extend Bianchi’s CSMA/CA analytical framework to UAMANETs, enabling a quantitative characterization of how spectrum resource allocation affects AMIE through link activation probability, transmission interruption, and end-to-end hop count. Building on the derived analytical insights, we further develop a soft centralized resource management framework, in which an existing MSF-PSO algorithm is employed as a numerical solver to optimize resource allocation under implicit MAC-layer coupling constraints. Numerical results demonstrate that, compared with conventional IEEE 802.11p spectrum resource settings, the proposed framework can achieve substantial AMIE improvements under representative network configurations.

## 1. Introduction

Mobile Ad Hoc Networks (MANETs) have attracted significant attention across military, industrial, and agricultural applications, leading to several specialized subclasses, including Flying Ad Hoc Networks (FANETs), Vehicular Ad Hoc Networks (VANETs), and Battlefield MANETs. A defining characteristic of MANETs is the absence of centralized infrastructure, which precludes centralized control entities such as Wi-Fi access points [1]. Consequently, nodes must autonomously adapt to dynamic network topologies and make independent operational decisions. This decentralized architecture has motivated the extensive adoption of distributed protocols for channel access, route establishment, and other core network functions [2].

In recent years, rapid advances in Unmanned Aerial Vehicle (UAV) technology have established UAVs as versatile communication platforms, offering flexible deployment, line-of-sight (LoS) connectivity, and robust adaptability to dynamic network conditions [3]. The enhanced Ground-to-UAV (G2U) channel enables ground nodes to communicate reliably with UAVs over extended distances without increasing transmission power. Consequently, integrating UAVs into MANETs to form UAV-assisted MANETs (UAMANETs) has emerged as a prominent research direction. Leveraging wide coverage and reliable point-to-point (P2P) links, UAVs have been extensively used as sink nodes in wireless sensor networks [4], airborne roadside units in VANETs [5], and high-efficiency relay nodes in various wireless networks [6]. In this work, we consider UAMANET scenarios in which UAVs act as relay nodes to support multi-hop communication among ground users, which represents a fundamental and widely studied UAV function. Existing research in this area has primarily focused on improving network performance through routing optimization and UAV mobility control.

From the routing perspective, highly dynamic UAMANET environments have motivated the development of routing protocols that can cope with frequent topology changes and intermittent connectivity. These protocols are commonly classified as topology-aware [7] or geographic-based. Topology-aware protocols, such as classic Dynamic Source Routing (DSR) and Zone Routing Protocol (ZRP), rely on detailed network state information and may incorporate learning-based enhancements, such as adaptive Q-learning [8], to improve routing reliability. However, maintaining accurate topology information incurs substantial communication, storage, and computational overhead, which limits scalability in dense or highly mobile networks. Geographic-based protocols, including Classic Greedy Perimeter Stateless Routing (GPSR) and Best-Link and 3D Perimeter forwarding Routing protocol (BLPR) [9], reduce overhead by exploiting node location information and have been further extended with UAV-assisted three-dimensional forwarding mechanisms. Their performance, however, degrades significantly when accurate location information is unavailable, such as in tunnels or urban canyon environments.

Beyond routing design, UAV trajectory planning plays a critical role in maintaining network connectivity [10], efficiently collecting information from ground-based wireless sensor networks [11] and reducing End-to-End (E2E) latency [12]. A variety of trajectory optimization methods have been proposed, including heuristic and optimization-based approaches such as Particle Swarm Optimization (PSO) [13] and convex optimization [14]. More recently, deep reinforcement learning techniques, including Deep Deterministic Policy Gradient (DDPG) [15] has been applied to enable adaptive UAV deployment in dynamic environments, supporting data collection, relaying, and secure communication.

In addition to routing and trajectory design, clustering has emerged as an effective strategy for organizing ground nodes into local groups in UAMANETs [16]. In cluster-based architectures, each cluster elects a cluster head (CH) to coordinate communications with UAVs on behalf of its cluster members (CMs), thereby improving routing efficiency and facilitating data aggregation [17]. For example, a cluster-based UAV-assisted VANET was proposed in [18], where UAVs relay data packets between vehicles in highway scenarios to reduce latency. Similarly, the clustering scheme in [19] selects cluster heads based on residual energy, mobility characteristics relative to UAV speed, and neighborhood density, highlighting the importance of clustering in improving network organization and performance.

In summary, although prior research has provided valuable insights into routing strategies, UAV deployment, clustering, and mobility control, it has largely emphasized performance metrics such as connectivity, delay, energy efficiency, and reliability. The fundamental issue of network capacity in UAMANETs operating remains insufficiently explored. Moreover, many existing solutions are scenario-specific and lack a unifying analytical framework. In contrast, capacity-oriented analysis offers a more fundamental and generalizable basis for understanding performance limits and for guiding the design of resource allocation and access mechanisms.

### 1.1. Motivation

From a capacity perspective, the seminal scaling-law analysis of Gupta and Kumar [20] shows that extending transmission range through long-distance relays reduces the number of simultaneously active transmitters at a higher order than the reduction in end-to-end hop count, ultimately leading to a decrease in asymptotic network capacity. This conclusion, however, is derived under idealized assumptions, including negligible transmission interruption probability and perfectly reliable links. In practical MANETs, contention-based medium access and unreliable multi-hop transmissions are unavoidable, and the achievable network load is often constrained by end-to-end transmission interruptions rather than by hop count alone. In this context, the highly reliable P2P links provided by UAVs offer a potential mechanism for improving network load capacity by reducing end-to-end transmission interruptions, which necessitates analysis tailored to the specific network environment.

As discussed earlier, MANETs typically rely on self-organizing channel access, which has led to the widespread adoption of IEEE 802.11 [21] MAC protocols [22]. In IEEE 802.11 networks, collision avoidance is primarily governed by the Distributed Coordination Function (DCF). However, as reported in [23], in DCF-based UAMANETs, G2U transmissions are considerably more vulnerable to hidden terminal effects, resulting in a substantially higher outage probability. As network load increases or the number of ground nodes within UAV coverage grows, this degradation in G2U reliability can diminish the performance benefits of UAV relaying, causing the UAMANET to gradually resemble a conventional MANET. To mitigate such interference, the widely adopted IEEE 802.11p-based Wireless Access in Vehicular Environments (WAVE) protocol assigns G2G and G2U/U2G transmissions to orthogonal Service Channels (SCHs). Despite this design, the channel-based resource allocation strategy of IEEE 802.11p exhibits two inherent limitations. First, transmission resources are allocated in fixed 10 MHz SCH units, which leads to coarse-grained partitioning and limited flexibility. Second, although network capacity remains a central bottleneck in UAMANET applications, IEEE 802.11p does not provide a systematic or theoretically grounded framework for resource allocation optimization from a capacity perspective.

To address these issues, this paper introduces the concept of Aggregate Multi-Hop Information Efficiency (AMIE) [24]. AMIE quantifies the effective amount of information successfully delivered per unit time, aggregated over all multi-hop transmission paths, while explicitly accounting for medium access contention, hop count, and end-to-end transmission reliability. Unlike conventional throughput or single-link capacity metrics, which typically focus on isolated links or assume reliable transmissions [25], AMIE captures the joint influence of routing decisions and MAC-layer access behavior on the overall information-carrying capability of UAMANETs under realistic contention and unreliable multi-hop conditions. Since these challenges are fundamentally related to resource allocation, the objective of this study is to maximize the AMIE of task clusters while satisfying the transmission requirements of the aerial backbone network through efficient resource allocation. In this work, G2G and G2U/U2G transmissions are assumed to operate over mutually orthogonal transmission resources, ensuring interference-free operation between link types. While this assumption is inspired by the orthogonal channel allocation concept of IEEE 802.11p, we do not restrict the analysis to actual SCHs. For analytical tractability, we introduce a finer-grained abstraction of transmission resources within each orthogonal domain, inspired by the Resource Unit concept of IEEE 802.11ax. This abstraction enables fine-grained optimization of resource allocation across different link types.

### 1.2. Outcome and Main Contributions

In this paper, we consider a representative UAMANET architecture in which a subset of UAVs forms stable task clusters with ground nodes, serving as relaying and aggregation points for intra-cluster communications. Simultaneously, these UAVs act as constituent nodes of an airborne backbone network. The spatial distribution of task clusters is modeled as a Poisson cluster process, while ground nodes within each cluster follow Poisson point processes (PPPs). Under this network model, task cluster capacity is characterized using the AMIE, which captures the joint effects of multi-hop routing, medium access contention, and transmission reliability. Using this framework, the impacts of key system parameters on AMIE, with particular emphasis on resource allocation schemes, are analytically and quantitatively characterized. The main contributions of this work are summarized as follows.

- Analysis of AMIE and Resource Allocation Scheme in UAMANET: Under the proposed network model and commonly used access schemes in current UAMANET, we extended Bianchi’s research on DCF and analyzed the impact of system parameters on AMIE. The results indicate that, resource allocation schemes exert multifaceted effects on AMIE under saturation; Specifically, increasing G2U transmission resources simultaneously reduces network link activation density, G2U transmission interruption probability, and the average E2E hop count, highlighting the inherent trade-offs in resource allocation. Moreover, factors such as access node density, carrier sensing range, and the probability of P2P transmission interruption—as influenced by the network environment and routing parameters—directly affect the convergence of the optimal resource allocation scheme.
- Resource Management Framework: Considering the decentralized nature of MANETs, we propose a soft-centralized resource allocation and management framework. In this framework, UAV nodes partition network resources into G2G and G2U transmission resources based on global network parameters, while ordinary ground nodes and ground nodes accessing UAVs perform distributed channel access within their allocated resources. Given that the current model lacks closed-form expressions for key parameters such as τ, we employ a MSF-PSO algorithm [26] to jointly optimize the resource allocation variables and access parameters.

From a methodological standpoint, the novelty of this work lies in the introduction of AMIE as a unified capacity metric and in the extension of Bianchi’s CSMA/CA analysis to UAMANETs by jointly incorporating contention-based MAC behavior, multi-hop routing, and E2E reliability. The remainder of this paper is organized as follows. Section 2 presents the system model and the adopted performance metric. Section 3 analyzes the differences in transmission and interference ranges among the three types of links in UAMANETs, namely G2G, G2U/U2G, and U2U. Section 4 investigates the key parameters affecting AMIE. Based on this analysis, Section 5 designs a resource allocation strategy to optimize AMIE in resource-constrained networks. Numerical and simulation results are provided in Section 6, and conclusions are drawn in Section 7.

## 2. System Model

### 2.1. Network Model

**Figure 1:**
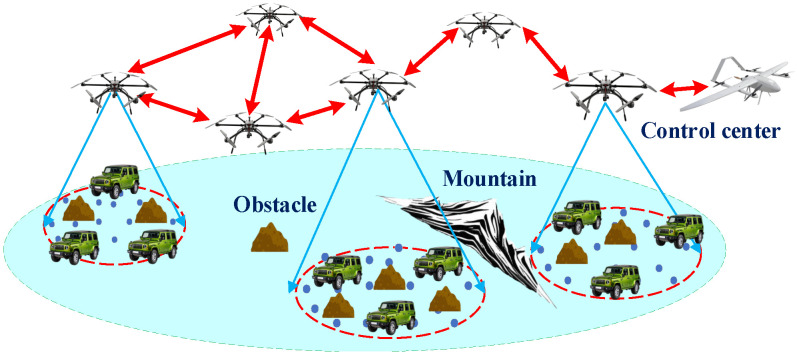
Network Model.

We consider a hybrid UAV-assisted network as shown in Figure 1, comprising $N_{u}$ UAVs and multiple sparsely distributed ground task clusters. A subset of UAVs, denoted by $n_{u}=N_{u}p$, serves permanently as cluster heads for specific ground clusters within their coverage areas. Each cluster-head UAV facilitates intra-cluster service transmissions and forwards aggregated traffic to the control center via the UAV backbone network. UAVs not designated as cluster heads operate exclusively within the backbone network, relaying aggregated traffic and maintaining overall network connectivity.

Within each task cluster, ground nodes are modeled as a homogeneous Poisson point process (HPPP) with density $\delta_{g}$. This modeling choice is widely adopted in MANET and the stochastic geometry literature to capture spatial randomness and node mobility while enabling tractable spatial averaging of interference and contention effects. Ground nodes are assumed to establish indirect connectivity through multi-hop transmissions, reflecting the self-organizing nature of infrastructure-less MANETs. ﻿

Traffic within each cluster is confined to communications among ground nodes belonging to the same cluster. This assumption is representative of task-driven UAMANET applications, where sensing, data aggregation, and local relaying are performed within a localized area before aggregated data are forwarded through the UAV backbone network. By focusing on intra-cluster traffic, the analysis captures the dominant contention and interference behaviors relevant to network load capacity.

All UAVs and ground nodes are assumed to employ identical transmission power and modulation and demodulation schemes. This assumption removes hardware-induced heterogeneity and allows the analysis to isolate the impact of MAC-layer contention, routing behavior, and resource-domain partitioning on overall network capacity. ﻿

To facilitate tractable analysis, task clusters are assumed to be spatially well separated, such that interference originating from nodes outside a typical cluster experiences substantial path loss attenuation. As a result, system performance is dominated by intra-cluster interference, which is the primary limiting factor under dense local contention. This assumption reflects practical task-oriented deployments and enables the analysis to focus on the dominant MAC-layer interactions without loss of generality. Common abbreviations used in this paper are shown in Table 1. ﻿

### 2.2. Channel Model

The large-scale channel power gain between a transmitting node *i* and a receiving node *j* is defined as

(1) $$h_{ij}=l\left(d_{ij}\right)$$

where $d_{ij}=\left|X_{i}-X_{j}\right|$ denotes the Euclidean distance between nodes *i* and *j*, and l(·) represents the distance-dependent path loss function. Accordingly, the received power at node *j* is given by $P_{r}^{j}=P_{i}h_{ij}$, where $P_{i}$ denotes the transmission power of node *i*. For different link types, the channel power gain is modeled as

(2) $$h_{ij}=\left\{\begin{array}{cc} d_{ij}^{-\alpha_{g}}, & G2G\;\mathrm{links},\\ d_{ij}^{-\alpha_{u}}, & U2U\;\mathrm{links},\\ d_{ij}^{-\alpha_{ug}}, & G2U/U2G\;\mathrm{links}. \end{array}\right.$$

**Figure 2:**
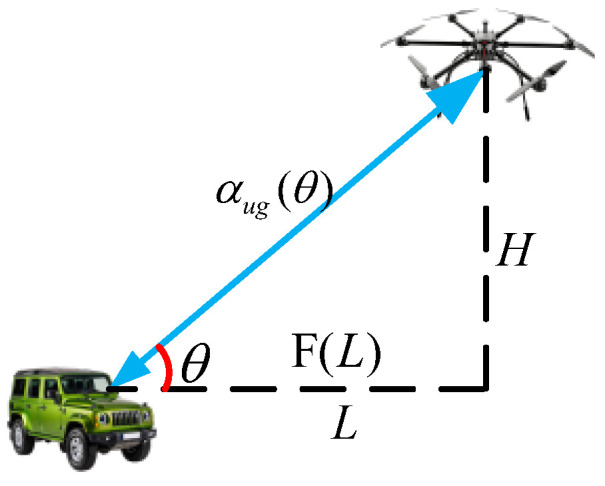
Ground to air transmission parameters.

A P2P transmission is considered successful if the received Signal-to-Interference-plus-Noise Ratio (SINR) satisfies

(3) $$\frac{P_{i}h_{ij}}{N_{0}+\sum_{k\in T\;k\neq i}P_{k}h_{kj}}⩾\beta$$

where $N_{0}$ denotes background noise power, T is the set of concurrently transmitting nodes, and β is the SINR threshold for successful decoding. For G2U/U2G links, the channel power gain depends on both the elevation angle and the UAV altitude. Under a fixed UAV altitude *H*, the channel gain can be expressed as

(4) $$h_{ug}\left(L\right)={\left(L^{2}+H^{2}\right)}^{-\frac{\alpha_{ug}\left(L/H\right)}{2}}$$

where *L* denotes the horizontal distance between the UAV and the ground node. To enable tractable analysis, as shown in Figure 2, an equivalent horizontal path loss exponent F(L) is introduced such that

(5) $${\left(L^{2}+H^{2}\right)}^{-\frac{\alpha_{ug}\left(L/H\right)}{2}}≜L^{-F(L)}.$$

In conventional UAMANETs, the path loss exponents typically satisfy $\alpha_{g}<F(\cdot )<\alpha_{u}$. Finally, for IEEE 802.11p operating over a bandwidth *b*, the corresponding physical-layer transmission rate is given by

(6) $$R\left(b\right)=\frac{b}{10\;\mathrm{MHz}}R_{p}\left(\beta \right)$$

where $R_{p}\left(\beta \right)$ denotes the achievable data rate under the SINR threshold β.

### 2.3. Performance Metric

As discussed earlier, the network capacity analysis in [20] is based on scaling laws and cannot fully capture the effects of network planning parameters and specific communication strategies on actual network capacity. To optimize transmission resource allocation, we adopt the AMIE [m·bits/(s·Hz·m^2^)] definition from [24]:

(7) $$A=C_{b}\lambda_{\mathrm{act}}{\left(1-P_{\mathrm{out}}\right)}^{\bar{h}}\bar{L}d$$

where $C_{b}=\log_{2}\left(1+\beta \right)$ denotes the P2P spectral efficiency, $\lambda_{\mathrm{act}}$ is the density of active links, $P_{\mathrm{out}}$ and ${\bar{L}}_{d}$ represent the P2P transmission outage probability and the average effective distance toward the destination per activated link, respectively. In the considered network, all nodes operate under a uniform modulation scheme with a common SNR threshold β, and the average distance $\bar{L}$ between source and destination nodes is fixed within a cluster. Considering heterogeneous link outage probabilities, AMIE can be reformulated as

(8) $$\bar{A}:=\frac{\bar{A}}{\bar{L}C_{b}}=\lambda_{\mathrm{act}}\bar{P}\bar{E}$$

where ${\bar{P}}_{s}$ and $\bar{E}={\bar{L}}_{d}/\bar{L}$ denote the average E2E successful transmission probability and average transmission efficiency of activated links, respectively.

## 3. Preliminary Analysis

**Figure 3:**
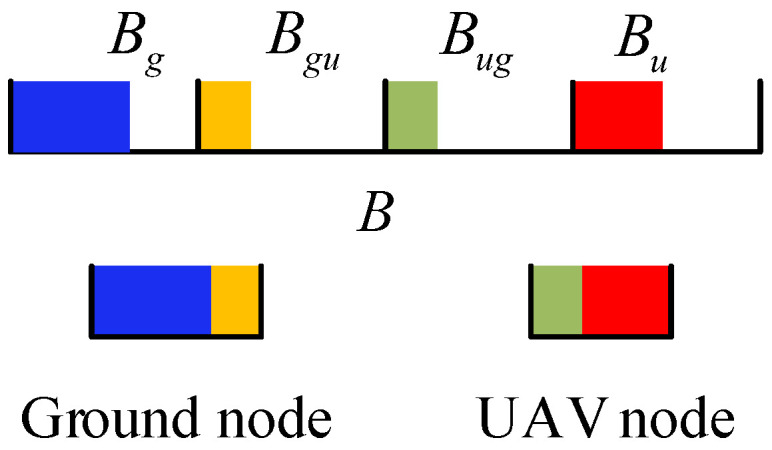
Resource management schemes.

As discussed in Section 1.1, this study focuses on resource allocation in UAMANETs based on Resource-Domain Partitioning (RDP) using finer-grained resource units. We further investigate how detailed resource segmentation affects network performance. As illustrated in Figure 3, under this strategy, the total resources are first divided into orthogonal domains according to link type, namely $B=B_{u}\cup B_{ug}\cup B_{g}\cup B_{gu}$, corresponding to U2U, U2G, G2G, and G2U transmissions. These resources are then allocated to nodes associated with each link category.

This paper analyzes the main Interference Boundaries (IBs) for different link types to determine the required protection radius around a receiver node for a given transmitter–receiver separation $|X_{i}-X_{j}|$. This range is defined as

(9) $$R_{I}=\max\left\{R_{i}:\frac{P_{i}h_{ij}}{N_{0}+P_{k}/R_{i}^{\alpha_{i}}}⩾\beta \right\}$$

where $R_{I}$ denotes the maximum allowable distance between a potential interfering node and the intended receiver such that a single interfering transmission does not reduce the received SINR below the threshold β. The parameters $\alpha_{t}$ and $\alpha_{i}$ represent the path-loss exponents of the intended link and the interfering link, respectively.

### 3.1. IBs of Various Links

For collision-free G2G transmission between ground nodes $C_{t}$ and $C_{r}$ separated by distance $r_{g}=\left|X_{i}-X_{j}\right|$, according to Equation (9), by neglecting the background noise power $N_{0}$, the IBs for the terrestrial and aerial networks can be expressed as $g_{g}\left(r_{g}\right)=\sqrt[{\alpha_{g}}]{\beta }r_{g}$, $u_{g}\left(r_{g}\right)=\sqrt[{F(u_{g})}]{\beta r_{g}^{\alpha_{g}}}$. That is, all ground nodes within distance $g_{g}$ and all UAVs within horizontal distance $u_{g}$ from $C_{r}$ must use transmission resources orthogonal to those of $C_{t}$. Similarly, we can obtain the IBs corresponding to G2U, U2G and U2U transmissions, which can be represented as $g_{gu}$, $u_{gu}$, $g_{ug}$, $u_{ug}$, etc. To avoid collisions between any pair of nodes within transmission range, the Edge Interference Boundaries (EIBs) are defined as $G_{g}=g_{g}\left(R_{g}\right)+R_{g}$, $G_{ug}=g_{ug}\left(R_{ug}\right)+R_{ug}$, etc. Under typical UAMANET parameters, the EIBs generally satisfy $G_{g}<G_{ug}<G_{gu}<G_{u}$ and $U_{g}<U_{ug}<U_{u}<U_{gu}$. In CSMA/CA, nodes mitigate collisions through carrier sensing (CS). Specifically, under an energy detection threshold ψ, a transmitting node prevents others within a radius $R_{s}:P_{i}R_{s}^{-\alpha }=\psi$ from initiating the transmission process. Based on the path loss exponents satisfying $\alpha_{g}<F<\alpha_{u}$, UAMANETs exhibit three CS radii $R_{s}^{g}<R_{s}^{ug}<R_{s}^{u}$. Nodes located in the set difference between the IB and the transmitter’s CS region act as hidden terminals. When G2G, G2U, U2G, and U2U transmissions share a common resource domain, ground nodes face inherent challenges. For G2G, the terrestrial EIB is $G_{g}$ with sensing range $R_{s}^{g}$. For G2U, the EIB expands to $G_{gu}$ while the sensing range remains unchanged. Therefore, compared to G2G, G2U will be more susceptible to hidden terminal issues.

### 3.2. RDP Network Model

Based on the preceding analysis, the RDP network model is clarified as follows:

- Resource partitioning: The total transmission resources are divided into $B=B_{u}+B_{g}+B_{ug}+B_{gu}$. Such resource-domain partitioning enables interference isolation among heterogeneous link types while preserving distributed channel access within each domain.
- Transceiver capabilities: Both ground nodes and UAVs are equipped with full transceiver functionality, enabling them to transmit and receive data over all resource domains in *B*. This capability ensures that routing and relaying decisions are not constrained by hardware limitations and enables a unified treatment of different link types.
- Access mechanisms for homogeneous links: U2U and G2G links, which connect homogeneous nodes, access the channels $B_{u}$ and $B_{g}$ using CSMA/CA. This design choice maintains consistency with IEEE 802.11-based UAMANET implementations and explicitly captures contention-driven channel access behavior among nodes with similar communication characteristics.
- Ground node access to UAV: Ground nodes attempt to access the UAV with probability $p_{g}$ using either the CSMA/CA access mechanism or CSMA/CA with RTS/CTS. The probabilistic access mechanism preserves the distributed nature of MANETs.
- Resource assignments principle: All homogeneous nodes are assigned identical access priorities under CSMA/CA. This assumption ensures fairness among nodes of the same type and enables a clear characterization of how resource partitioning and contention jointly affect network load capacity.

Overall, the adopted modeling assumptions balance analytical tractability and physical relevance. While certain idealizations are introduced, they enable a clear characterization of the fundamental capacity behavior of UAV-assisted MANETs under contention-based MAC access. Importantly, the qualitative insights obtained from the proposed framework remain applicable beyond the specific assumptions considered. ﻿

## 4. Optimal Resource Allocation

Combined with the preceding analysis, this section evaluates how different resource allocation schemes influence the activated link density, E2E hop count, and E2E outage probability, and further develops the corresponding resource-optimization strategies.

### 4.1. Active Link Density

For CSMA/CA, Bianchi [27] established the fundamental model for the transmission attempt probability τ of a saturated node in a random backoff slot, which is also adopted in this work. We further define the average probability that a node attempts transmission in an arbitrary time slot as $\varepsilon =P_{bo}\tau$, where $P_{bo}$ denotes the fraction of backoff slots along the time axis and is given by

(10) $$P_{bo}=\frac{(1-{\bar{P}}_{tr})\sigma }{\left(1-{\bar{P}}_{tr}\right)\sigma +{\bar{P}}_{tr}{\bar{P}}_{s}T_{s}+{\bar{P}}_{tr}\left(1-{\bar{P}}_{s}\right)T_{c}}.$$

Here, $T_{s}$ and $T_{c}$ denote the durations of busy slots corresponding to successful transmission and collision, respectively, σ is the duration of an idle slot, ${\bar{P}}_{tr}$ characterizes the occurrence of channel activity, while ${\bar{P}}_{s}$ quantifies the likelihood that such activity corresponds to a collision-free transmission. Accordingly, the product ${\bar{P}}_{tr}{\bar{P}}_{s}$ represents the probability that a node in the channel transmits without collision in a random backoff slot. Physically, $P_{bo}$ represents the proportion of time during which the channel remains idle and the backoff counter can be decremented. It captures the time-domain discrepancy between backoff slots and actual channel slots caused by variable busy periods, thereby serving as a scaling factor that maps the per-backoff-slot transmission attempt probability τ to an effective transmission attempt probability in an arbitrary time slot. For G2G transmissions, ${\bar{P}}_{s}$ and $P_{tr}$ can be expressed as

(11) $$\begin{array}{cc} {\bar{P}}_{s}^{g}\left(L\right) & =\sum_{x\in S(R_{s}^{g},L)}\frac{\tau_{g}(|x|)[1-{{\bar{P}}_{c}}^{g}\left(|x|)\right]}{P_{tr}^{g}\left(|x|\right)} \end{array}$$

(12) $$\begin{array}{cc} P_{tr}^{g}\left(L\right) & =1-\prod_{x\in S(R_{s}^{g},L)}\left(1-{\bar{\tau }}_{g}\left(|x|\right)\right). \end{array}$$

Similarly, for G2U transmissions, ${\bar{P}}_{s}$ and $P_{tr}$ can be expressed as

(13) $$\begin{array}{cc} {\bar{P}}_{s}^{gu}\left(L\right) & =\sum_{g\in S(R_{s}^{g},L)}\frac{\tau_{gu}(|x|)[1-{{\bar{P}}_{c}}^{gu}\left(|x|)\right]}{P_{tr}^{gu}\left(|x|\right)} \end{array}$$

(14) $$\begin{array}{cc} P_{tr}^{gu}\left(L\right) & =1-\prod_{g\in S(R_{s}^{g},L)}\left(1-{\bar{\tau }}_{gu}\left(|x|\right)\right). \end{array}$$

For G2U transmission using RTS/CTS, ${\bar{P}}_{s}$ and $P_{tr}$ can be represented as

(15) $$\begin{array}{cc} {\bar{P}}_{s}^{gu}\left(L\right) & =\sum_{g\in N_{g}}\frac{\tau_{gu}(|g|)[1-{\bar{P}}_{c}^{ug}\left(|g|)\right]}{P_{tr}^{gu}\left(|g|\right)} \end{array}$$

(16) $$P_{tr}^{gu}\left(L\right)=1-\prod_{g\in S(R_{s}^{g},L)}\left(1-{\bar{\tau }}_{gu}\left(|g|\right)\right)+\sum_{g\in (N_{g}\setminus S\left(R_{s}^{g},L\right))}\tau_{gu}\left(|g|\right)\left(1-{\bar{P}}_{c}^{ug}\left(|g|\right)\right).$$

Here, ${\bar{\tau }}_{g}\left(|x|\right)$ and ${\bar{\tau }}_{gu}\left(|g|\right)$ denote the spatially averaged transmission attempt probabilities of a node located at distances |x| and |g| from the network center for G2G and G2U communications, respectively, while ${\bar{P}}_{c}^{g}$ and ${\bar{P}}_{c}^{ug}$ represent the corresponding collision probabilities of G2G and G2U links. The terms $\tau \left(\cdot \right)[1-P_{c}\left(\cdot \right)]$ and 1−τ(·) denote the probabilities that a node at the corresponding position successfully transmits and remains silent in a random backoff slot, respectively. The region $S(R_{s}^{g},L)$ denotes the effective channel sensing region of a node located at distance *L* from the network center and is defined as the intersection between the carrier sensing region of radius $R_{s}^{g}$, centered at the node, and the overall network coverage area. It is noted that when G2U transmission employs the RTS/CTS mechanism, the CTS response from the UAV triggers virtual carrier sensing and freezes the backoff counters of all gateway nodes within its coverage area. As a result, once a CTS is successfully received, the subsequent successful transmission occupies the channel for a duration $T_{s}$ that is perceived as a busy period by all such nodes. This effect is therefore incorporated into the evaluation of both $P_{s}$ and $P_{tr}$ as a contribution to channel busy probability associated with successful transmissions. Moreover, for G2G transmissions, *x* denotes a regular ground node, whereas for G2U transmissions, *g* denotes a gateway node. The detailed derivation of $P_{c}$ is given in Equation (22).

In the multi-hop network considered in this paper, nodes experience heterogeneous interference conditions due to spatial separation. Consequently, even when the MAC parameters are fixed, the τ of each node is still directly influenced by its conditional collision probability $P_{c}$, which varies with node location and transmission distance. Therefore, by substituting the location and distance dependent collision probability $P_{c}\left(L,s\right)$ into Bianchi’s classic formula for τ [27], we can express the τ associated with a node at a distance of *L* from the network center and a P2P transmission distance of *s* as

(17) $$\tau \left(L,s\right)=\frac{2\left(1-2P_{c}\left(\tau ,L,s\right)\right)}{\left(a+b\right)}$$

where $a=\left(W_{0}+1\right)\left(1-2P_{c}\left(\tau ,L,s\right)\right)$, $b=W_{0}P_{c}\left(\tau ,L,s\right)\left(1-{\left(2P_{c}\left(\tau ,L,s\right)\right)}^{m}\right)$. Based on this formulation, the location-dependent spatially averaged τ can be expressed as ${\bar{\tau }}_{g}\left(L\right)=\int_{0}^{R_{T}}{\bar{\tau }}_{g}\left(L,s\right)f\left(s\right)ds$, ${\bar{\tau }}_{gu}\left(L\right)=2\left(1-2P_{c}\left(\tau ,L\right)\right)/\left(a+b\right)$. Here, $R_{T}$ and *s* denote the P2P transmission range and the corresponding transmission distance, respectively, while f(s) is the probability density function determined by the adopted routing strategy. For G2G transmissions, once the routing policy is specified, the distribution f(s) is known. Consequently, the transmission attempt probability τ depends solely on the node’s spatial location *L*, with $\bar{\tau }=\int_{0}^{R_{ug}}\bar{\tau }\left(L\right)dL$. Accordingly, under conventional CSMA/CA, the average transmission attempt probability is given by ${\bar{\varepsilon }}_{cs}=\bar{\tau }P_{bo}$. For CSMA/CA with RTS/CTS, a collision occurring during the RTS phase prevents subsequent data transmission. Therefore, the probability that a node attempts to transmit a data packet in a random backoff slot is $\tau (1-P_{c})$, leading to ${\bar{\varepsilon }}_{rt}=\bar{\tau }\left(1-P_{c}\right)P_{bo}$.

Based on the above analysis, for G2G transmission employing CSMA/CA without RTS/CTS under saturated conditions, the active link density is given by ${\bar{\lambda }}_{g}={\bar{\tau }}_{g}P_{bo}\delta_{g}$. For G2U transmission, when ground nodes access the UAV using CSMA/CA without RTS/CTS, the corresponding active link density is ${\bar{\lambda }}_{gu}={\bar{\tau }}_{gu}P_{bo}\delta_{g}p_{g}.$ When CSMA/CA with RTS/CTS is adopted for G2U access, the active link density becomes ${\bar{\lambda }}_{gu}^{\prime }={\bar{\tau }}_{gu}\left(1-{\bar{P}}_{c}\right)P_{bo}\delta_{g}p_{g}.$ For U2G, since the transmission resources used by UAV cannot be reused by any other node within the task cluster, its the active link density is ${\bar{\lambda }}_{ug}=1/\left(\pi R_{ug}^{2}T_{s}\right).$ It should be noted that when each UAV acting as a cluster head is allocated $b_{ug}$ transmission resources, the total transmission resources consumed by the U2G links within the network is $B_{ug}=b_{ug}d_{u}$, where $d_{u}$ denotes the average number of other UAVs that also serve as cluster heads and lie within the $U_{ug}$ range of any given cluster-head UAV. In conclusion, we can obtain that, within a task cluster

(18) $$\begin{array}{c} {\bar{\lambda }}_{act}=\frac{B_{g}\lambda_{g}+b_{ug}\lambda_{ug}+B_{gu}\lambda_{gu}}{B}. \end{array}$$

In summary, besides the resource allocation scheme, the active link density $\lambda_{act}$ is jointly determined by the MAC parameters, the P2P transmission collision probability $P_{c}$, and the probability $p_{g}$ that a ground node accesses the UAV as a gateway.

### 4.2. Transmission Efficiency Analysis

**Figure 4:**
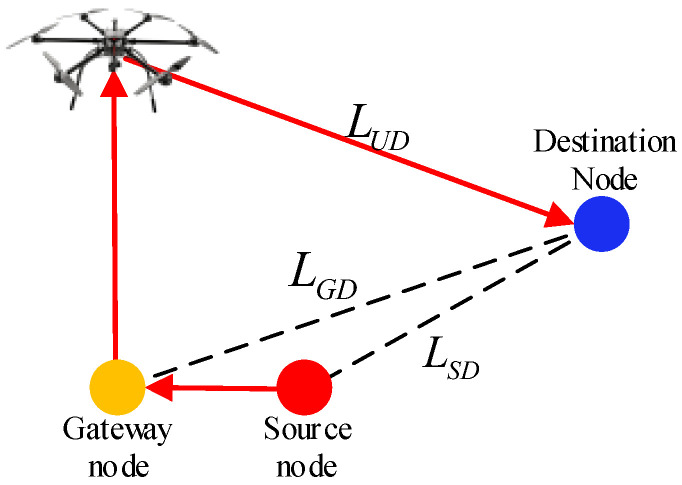
Route through the UAV in LAg.

Regarding the average transmission efficiency of activated links, data delivery in UAMANETs operates over a hybrid routing structure consisting of G2G, G2U, and U2G links. As illustrated in Figure 4, when the relationship $L_{UD}>L_{GD}>L_{SD}$ holds, the effective forward distances along both the source–gateway and gateway–UAV segments become negative. Under such conditions, defining transmission efficiency solely based on effective distance results in an overestimation of $|{\bar{R}}_{u}|$ and an underestimation of $|{\bar{R}}_{g}|$. To avoid this distortion, the average transmission efficiency of UAMANETs is defined as

(19) $$\bar{E}=\frac{B_{g}\lambda_{g}}{B{\bar{\lambda }}_{act}}\left[\frac{p_{u}}{{\bar{h}}_{u}}+\frac{1-p_{u}}{{\bar{h}}_{g}}\right]+\frac{b_{ug}\lambda_{ug}+B_{gu}\lambda_{gu}}{{\bar{h}}_{u}B{\bar{\lambda }}_{act}}.$$

In this expression, ${\bar{h}}_{u}$ and ${\bar{h}}_{g}$ denote the average hop counts of packets delivered via UAV-assisted paths and purely terrestrial paths, respectively. The parameter $p_{u}$ denotes the fraction of G2G transmission resources consumed by the average traffic load generated by UAV-assisted relay services on the terrestrial network. It can be defined as

(20) $$p_{u}=\frac{T[\mu_{u}]}{N_{g}{\bar{\varepsilon }}_{cs}}$$

where $T[\mu_{u}]$ denotes the number of transmission attempts associated with traffic relayed by the UAV on the terrestrial network.

For ${\bar{h}}_{u}$, in the RDP network, when traffic is relayed through the UAV, each packet is first forwarded to a gateway node, which subsequently transmits it to the UAV. Assuming that each ground node independently becomes a gateway with probability $p_{g}\leq 1$. The average number of E2E hops via the UAV is ${\bar{h}}_{u}=2+\bar{\Delta }$, where $\bar{\Delta }\;=\sum iPr(\Delta =i)$ and

(21) $$\begin{array}{cc} Pr(\Delta =i) & =\left\{\begin{array}{c} p_{g},\;i=0\\ \left(1-p_{g}\right)\left(1-\exp\left(-p_{g}\delta_{g}\bar{S}\left(R_{1}\right)\right)\right),i=1\\ \left(1-p_{g}\right)\exp\left(-p_{g}\delta_{g}\bar{S}\left(R_{i-1}\right)\right)\times \left(1-\exp\left(-p_{g}\delta_{g}\bar{S}\left(R_{i}\right)\right)\right),\;i>1 \end{array}\right. \end{array}$$

Here, $R_{i}=R_{g}+\left(i-1\right)\left|{\bar{R}}_{g}\right|$, where $\left|{\bar{R}}_{g}\right|$ denotes the average effective distance that a packet advances toward its destination in a single G2G transmission. This quantity depends on both the transmission range and the adopted routing strategy. For the derivation of ${\bar{h}}_{g}$, we assume that the UAV provides services independently and randomly over the network area. As a result, the original routing hop distribution of ground nodes remains unchanged. Under this assumption, the average number of G2G hops is given by ${\bar{h}}_{g}=\bar{L}/|R_{g}|$.

In summary, the average transmission efficiency of active links $\bar{E}$, is directly influenced by the average proportion of active G2G services that are relayed through the UAV $p_{u}$, as well as by the average E2E hop counts of UAV-assisted transmissions ${\bar{h}}_{u}$, and those completed entirely through ground nodes ${\bar{h}}_{g}$. The value of ${\bar{h}}_{u}$ is primarily determined by the gateway node density $\delta_{g}p_{g}$. The parameter $p_{u}$ will be discussed in detail in Section 4.3.

### 4.3. E2E Successful Transmission Probability Analysis

Regarding the success probability of P2P transmissions, this paper considers two primary sources of transmission failure: failures caused by the wireless propagation environment and node mobility, and failures induced by the MAC protocol. The effects of channel conditions and mobility are not analyzed in further detail. Instead, we introduce weighting factors $P_{eu}$ and $P_{eg}$ to represent the success probabilities of U2G and G2G transmissions, respectively, under environmental and mobility effects. Based on existing studies on U2G/G2U and G2G channels [28], the higher LoS probability and larger communication range of U2G/G2U links imply that $P_{eu}>P_{eg}$.

Regarding transmission failures induced by the MAC layer, U2G links employ conflict-free resource allocation. As a result, MAC-layer contention does not cause transmission failures on U2G links. In contrast, when a ground node located at a distance *L* from the network center transmits to another ground node at a distance *s* using CSMA/CA, the transmission success probability can be expressed as

(22) $$\begin{array}{cc} P_{m}^{g}\left(L,s\right) & =\prod_{x\in S\left(R_{s}^{g},L\right)\setminus \left\{x_{0}\right\}}(1-\tau_{g}\left(||x||)\right)\times \prod_{x\in (I_{g}\left(L,s\right)\setminus S\left(R_{s}^{g},L\right))}{(1-\tau_{g}\left(||x||)\right)}^{T_{v}} \end{array}$$

where $I_{g}\left(s\right)=\min\left(\pi R_{ug}^{2},S\left(g_{g}\left(s\right),L\right)\right)$. For a G2U link with a transmission distance of *s* using CSMA/CA, the success probability is given by

(23) $$\begin{array}{cc} P_{m}^{u}\left(s\right) & =\underset{P_{l}}{\underset{︸}{\prod_{x\in S\left(R_{s}^{g},s\right)\setminus \left\{x_{0}\right\}}\left(1-\tau_{gu}\left(||x||\right)\right)}}\times \underset{Hidden\;Terminal\;Free}{\underset{︸}{\prod_{x\in (S\left(g_{gu}\left(s\right),0\right)\setminus S\left(R_{s}^{g},s\right))}{\left(1-\tau_{gu}\left(||x||\right)\right)}^{T_{v}}}} \end{array}$$

The average values of $P_{m}^{u}\left(L\right)$ and $P_{m}^{g}\left(L,s\right)$ can be derived using the Probability Generating Functional (PGFL). In these expressions, $1-P_{l}$ represents the probability that a transmission fails due to multiple nodes within the CS range completing their backoff simultaneously. The parameter $T_{v}$ denotes the vulnerable period during which a P2P transmission may be disrupted by interference from hidden terminals.

**Figure 5:**
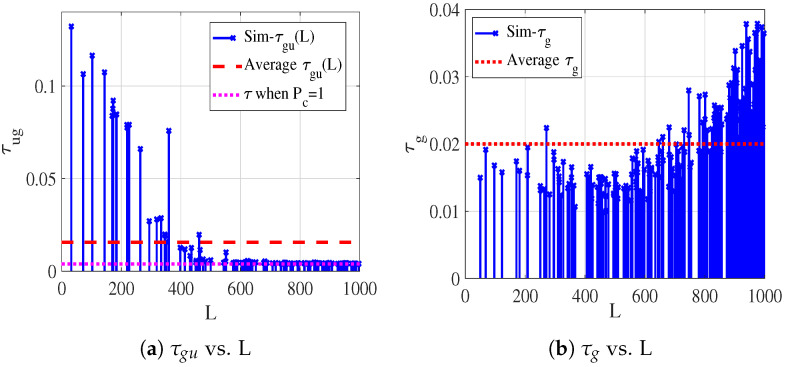
The corresponding $\tau_{ug}$ and $\tau_{g}$ to the nodes at distance L from the network center.

However, as illustrated in Figure 5, under the simulation parameters in Table 2, we evaluate $\tau_{g}$ and $\tau_{ug}$ for ground nodes at a horizontal distance *L* from the UAV, with $p_{g}\delta_{g}=1.5\times 10^{-6}\;{\mathrm{nodes}/m}^{2}$ and $T_{s}=52$ slots. As shown in Figure 5b, increasing *L* reduces $S(R_{s}^{g},L)$ due to edge effects, which decreases the collision probability and increases τ. However, as shown in Figure 5a, when L>400 m, τ drops to 0.0039 with $P_{c}=1$, indicating that G2U transmission becomes infeasible beyond this distance. As $p_{g}$ and $T_{s}$ continue to increase, this situation worsens. Consequently, CSMA/CA without RTS/CTS is not suitable for G2U transmission. When CSMA/CA with the RTS/CTS mechanism is employed, the G2U transmission success probability follows Equation (23), with $T_{v}$ replaced by $T_{RTS}$. Since $T_{RTS}\ll T_{v}$, the RTS/CTS mechanism substantially improves $P_{mu}$. Based on this, we can obtain the average success probability of G2U and G2G links under the MAC protocol as ${\bar{P}}_{m}^{g}\left(L\right)=\int_{0}^{R_{g}}\frac{2L}{R_{ug}^{2}}P_{m}^{g}\left(L,s\right)f\left(s\right)ds$. Within the RDP network considered in this paper, the collision probability of a G2G transmission is given by ${\bar{P}}_{c}^{g}\left(L\right)=1-{\bar{P}}_{m}^{g}\left(L\right)P_{eg}$, while for G2U transmission, the collision probability is ${\bar{P}}_{c}^{u}\left(L\right)=1-{\bar{P}}_{m}^{u}\left(L\right)P_{eu}$. Accordingly, we define ${\bar{P}}_{tg}={\bar{P}}_{m}^{g}\left(L\right)P_{eg}$ and ${\bar{P}}_{tu}={\bar{P}}_{m}^{u}\left(L\right)P_{eu}$ as the probabilities of successful G2G and G2U transmissions. The corresponding average E2E success probabilities are given by   

(24) $$\begin{array}{cc} \bar{P}= & \frac{B_{g}{\bar{\lambda }}_{g}}{B{\bar{\lambda }}_{act}}\left[p_{u}P_{tu}P_{eu}E\left[P_{tg}^{\Delta }\right]+\left(1-p_{u}\right)E\left[P_{tg}^{h_{g}}\right]\right]+\frac{b_{ug}\lambda_{ug}+B_{gu}\lambda_{gu}}{B{\bar{\lambda }}_{act}}P_{tu}P_{eu}E\left[P_{tg}^{\Delta }\right] \end{array}$$

where $P_{tu}P_{eu}E\left[P_{sg}^{\Delta }\right]$ and $E[P_{sg}^{{\bar{h}}_{g}}]$ are the average success probabilities of services transmitted with UAV assistance and those transmitted entirely in terrestrial networks, respectively. For $T[\mu_{u}]$ in $p_{u}$, under the considered scenario where G2G traffic is dominant, $\mu_{u}$ is constrained by the uplink and downlink bottlenecks at the UAV, that is $\mu_{u}=\min\left(C_{up},C_{dn}\right)$. When $b_{ug}+B_{gu}$ is fixed, $\mu_{u}$ is maximized when $C_{dn}=C_{up}$. For ease of analysis, we assume that the packet length transmitted within the network is E[P]. Based on this assumption, the UAV uplink and downlink capabilities can be expressed as $C_{dn}=\left(1-\eta \right)R\left(b_{ug}\right)P_{eu}$ and

(25) $$C_{up}=\frac{P_{tr}^{gu}{\bar{P}}_{s}^{gu}E\left[P\right]}{\left(1-P_{tr}^{gu}\right)\sigma +P_{tr}^{gu}{\bar{P}}_{s}^{gu}T_{s}+P_{tr}^{gu}\left(1-{\bar{P}}_{s}^{gu}\right)T_{c}}.$$

Here, η denotes the comprehensive MAC/PHY-layer overhead ratio, which accounts for the preamble, control signaling, acknowledgments, and protection intervals. Under CSMA/CA with RTS/CTS, $T_{c}$ and $T_{s}$ can be written as $T_{c}=T_{RTS}+DIFS$ and $T_{s}=T_{RTS}+SIFS+T_{CTS}+SIFS+T_{data}+SIFS+T_{ACK}+DIFS$, where DIFS and SIFS denote the Distributed InterFrame Space and Short InterFrame Space in CSMA/CA, respectively. The packet transmission time $T_{\mathrm{data}}$ is given by $T_{\mathrm{data}}=\left(E[P]+\mathrm{MAC}\;\mathrm{head}\right)/R(B_{gu})$. Therefore, when the UAV operates under saturated conditions, the total number of transmission attempts generated in the ground network can be expressed as $T\left[\mu_{u}\right]=\frac{C_{up}\sigma \bar{\Delta }}{E\left[P\right]P_{tg}}$. Substituting this expression into Equation (20), together with Equation (25), yields the final expression for $p_{u}$. In summary, the average E2E transmission success probability is not only influenced by the previously discussed hop counts, but also by the P2P transmission success probabilities $P_{sg}$ and $P_{su}$. Moreover, the parameter $p_{u}$ used in the preceding analysis is jointly determined by the spectrum resource allocation scheme and the values of $P_{sg}$ and $P_{su}$.

## 5. Resource Optimization

Based on the preceding analysis, the problem of maximizing AMIE can be modeled as an optimization problem of resource allocation schemes and $p_{g}$ parameters. In this section, we model and simplify the above problem using a real network and design a resource allocation optimization method for the UAMANET environment considered in this paper.

### 5.1. Problem Modeling and Simplification

Considering a practical network, the total available frequency resource is denoted by *B*, with a minimum allocation unit *b*, such that B=Nb. Each UAV reserves $b_{uu}=m_{u}b$ resources for U2U transmission to maintain backbone connectivity. Under conventional network settings, the following system parameters are assumed to be known: ground node density $\delta_{g}$; G2G transmission range $R_{g}$ and carrier sensing range $R_{s}^{g}$; and G2U transmission range $R_{ug}$ and carrier sensing range $R_{s}^{ug}$. Based on the preceding analysis, the three components of AMIE, namely the activated link density $\lambda_{act}$, transmission efficiency $\bar{E}$, and E2E transmission success probability $\bar{P}$, are given in Equations (18), (19) and (24). These quantities can be expressed as $\lambda_{act}\left(B_{g},b_{ug},B_{gu},p_{g},d_{u}\right)$, $\bar{E}\left(B_{g},b_{ug},B_{gu},p_{g},{\bar{h}}_{g}\right)$, and $\bar{P}\left(B_{g},b_{ug},B_{gu},p_{g},{\bar{h}}_{g},P_{eg},P_{eu},f_{ug}\left(L\right),f_{g}\left(L\right)\right)$, respectively. The parameters $d_{u}$, ${\bar{h}}_{g}$, $P_{eg}$, $P_{eu}$, and $p_{g}$ depend on the network environment and routing strategy. Since they are independent of the spectrum resource management problem addressed in this paper, they are treated as fixed network characteristics during optimization. As a result, maximizing AMIE reduces to a three-variable optimization problem. Specifically, letting $B_{g}=c_{g}b$, $B_{ug}=c_{ug}b$, and $B_{gu}=c_{gu}b$, the resource allocation problem can be formulated as

(26) $$\begin{array}{c} \max_{\left\{c_{g},c_{ug},c_{gu}\right\}}A\left(\underset{\mathrm{PPs}}{\underset{︸}{B,\delta_{g},R_{g},R_{s}^{g},R_{ug},R_{s}^{ug},N_{u},p_{g},}}\underset{\mathrm{SPs}}{\underset{︸}{f_{g}\left(L\right),d_{u},P_{eu},P_{eg}}}\right) \end{array}$$

(27) $$\begin{array}{cc} s.t.\;\; & b\left(N_{u}m_{u}+c_{g}+c_{gu}+c_{ug}d_{u}\right)\leq B \end{array}$$

(28) $$\begin{array}{cc} \; & c_{ug},c_{g},c_{gu}\in Z^{+} \end{array}$$

(29) $$\begin{array}{cc} \; & C_{dn}=C_{up},\;p_{g}\leq 1,\;p_{u}\leq 1 \end{array}$$

where PPs denote preset parameters, whereas SPs denote statistical parameters. In contrast to PPs, SPs need to be obtained statistically from the network. Based on this, we designed the following resource optimization method for ground mission clusters.

### 5.2. Soft-Centralized Spectrum Resource Management Methods

In UAMANETs, the wide transmission and reception range of UAVs enables them to obtain network information with relatively low overhead and to broadcast resource allocation plans to a large number of nodes. This capability makes UAVs well suited to function as resource allocation entities. Accordingly, in the network environment considered in this paper, the UAV operates as a soft centralized controller that collects the required SP information, performs the optimization process, and subsequently broadcasts the resulting resource allocation plan to the ground task cluster. Specifically, resource allocation will be based on the following three phases:

Initialization and parameter acquisition: As described above, the UAV only needs to obtain the required SPs to perform resource optimization. To this end, the network is first initialized using a preset configuration $c_{g}^{0},c_{ug}^{0},c_{gu}^{0}$, which is computed by substituting typical values of $d_{u}$, ${\bar{h}}_{g}$, $P_{eu}$, and $P_{eg}$ into Equation (26). This preset configuration provides an operational baseline before parameter-aware optimization begins. After initialization, the UAV continuously collects the actual system parameters during network operation and subsequently executes the optimization procedure. For the number of other UAVs acting as cluster heads that may interfere with U2G transmission, denoted by $d_{u}$, the UAV leverages the difference in the coverage ranges of U2G and U2U transmissions. Since $U_{ug}<R_{u}$, the UAV can obtain the number of interfering cluster-head UAVs within $U_{ug}$ through signaling exchanges inside the backbone network. For $P_{eu}$, the UAV can directly estimate it by statistically evaluating the U2G transmission success rate. For $P_{eg}$, as indicated by Equation (22) and the relation $P_{sg}={\bar{P}}_{mg}P_{eg}$, $P_{eg}$ can be derived at the ground gateway node through statistical measurement of the average G2G transmission success probability. Similarly, ${\bar{h}}_{g}$ and $f_{g}\left(L\right)$ can be obtained by the ground gateway node by statistically analyzing the average end-to-end hop count of packets forwarded only through the ground network and the distance distribution of G2U and G2G transmissions. By embedding $P_{tg}$, ${\bar{h}}_{g}$ and $f_{g}\left(L\right)$ in its uplink transmissions to the UAV, the ground gateway provides the UAV with all the parameters required for resource optimization.

Optimization problem solving: Since τ in CSMA/CA does not admit a closed-form expression and the convexity of AMIE cannot be analytically proven, we employ a heuristic optimization method. In this work, the MSF-PSO algorithm [26] is employed to jointly optimize the resource allocation variables $c_{g},c_{ug},c_{gu}$ with AMIE as the objective function. MSF-PSO extends conventional Particle Swarm Optimization (PSO) by incorporating a multi-swarm structure and fitness-feedback mechanisms, which improve robustness against local optima in non-convex search spaces, while requiring fewer algorithmic control parameters than Genetic Algorithms (GA) or Differential Evolution (DE). These properties make MSF-PSO a computationally efficient and well-suited optimizer for the UAMANET resource allocation problem studied in this paper. Given the discrete nature of the decision variables and the coupling effect introduced by τ, MSF-PSO provides an effective means to explore the feasible solution space with moderate computational complexity.

Deploy optimization results: After completing the optimization, the UAV maps the optimized parameters $\left[c_{ug},c_{g},c_{gu}\right]$ to the corresponding frequency bands in *B*. The UAV then broadcasts the resource allocation configuration. Upon receiving this broadcast, each node switches to the designated frequency band and performs transmissions according to the published allocation scheme.

## 6. Evaluation

This section first validates the proposed analytical models for link activation density, transmission efficiency, and E2E transmission success probability. It then compares AMIE under different resource allocation schemes, thereby demonstrating the necessity of adopting the MSFPSO algorithm for resource allocation optimization through comparison with the default IEEE 802.11p scheme. Model validation is performed via Monte Carlo simulations implemented in MATLAB 2022a, with all results averaged over 50 independent runs using different random seeds generated by the rng() function.

We consider a representative UAMANET scenario in which UAVs form stable task clusters to support communications among mobile ground nodes. All ground nodes operate under saturated traffic conditions, with non-empty transmission queues at all times. Ground nodes are generated according to a conditioned PPP, excluding isolated nodes to ensure network connectivity. The total system bandwidth, ground node density, and access mechanisms are summarized in Table 2. To ensure a fair comparison with IEEE 802.11p, the bandwidth consumption of U2U transmissions is not explicitly modeled, and the total system bandwidth is fixed as $B=B_{g}+B_{ug}+B_{gu}=2\times \mathrm{SCH}=20$ MHz. Consistent with IEEE 802.11p, control signaling is carried over the control channel, while only network-layer data packets are transmitted over the data channels. For analytical simplicity, all data packets are assumed to have a fixed payload size of 5000 bits.

The parameters in Table 2 serve as default values throughout the evaluation. When examining the impact of a specific parameter, all others are held constant. The communication ranges of ground nodes and UAVs follow [29], and the SINR threshold β and MAC-layer data rate at B=10 MHz are set according to the IEEE 802.11p bandwidth–SINR relationship.

### 6.1. Derived Models Validated

This subsection validates the proposed analytical models in terms of activated link density, transmission efficiency, and E2E transmission success probability. We begin with the validation of the activated link density.

#### 6.1.1. Activated Link Density

**Figure 6:**
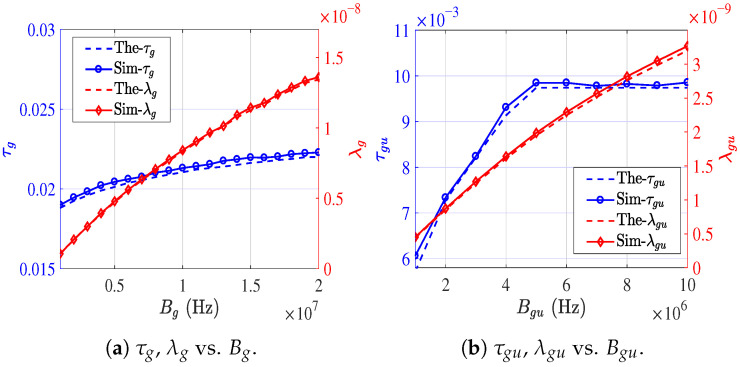
The impact of resource allocation on τ and link activation density.

To verify the average activated link density in Equation (18), we separately validate the analytical models for $\lambda_{g}$ and $\lambda_{ug}$. Focusing on the resource allocation problem considered in this paper and adopting the default parameters listed in Table 2, Figure 6a and Figure 6b illustrate the simulated (Sim-) and theoretical (The-) results of $\tau_{g}$, $\tau_{ug}$, $\lambda_{g}$, and $\lambda_{gu}$ as functions of $B_{g}$ and $B_{gu}$, respectively.

**Figure 7:**
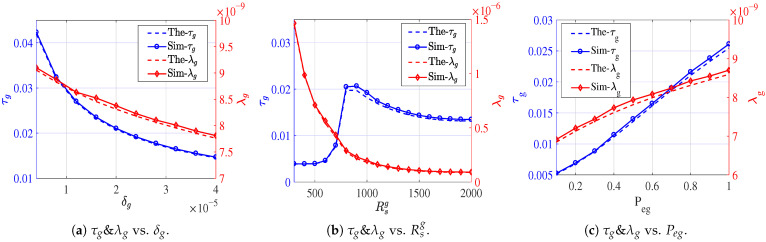
The corresponding $\tau_{g}\&\lambda_{g}$ under different parameters.

Sim-$\tau_{g}$ is obtained by averaging over ten independent Monte Carlo runs, while the theoretical value of $\tau_{g}$ is computed using the mean number of carrier-sensing nodes $\bar{S}\left(R_{s}^{g}\right)$. Since ${\left(1-\tau \right)}^{E[S]}<E\left[{\left(1-\tau \right)}^{S}\right]$, the theoretical analysis slightly underestimates $\tau_{g}$. Nevertheless, the maximum deviation remains below 3%, which is considered acceptable for analytical approximation. For the G2U activated link density under CSMA/CA with RTS/CTS, and to maintain consistency with IEEE 802.11p, control frames including RTS, CTS, and ACK are transmitted using a fixed low-rate MCS of 3 Mbps. Specifically, when $B_{gu}>5$ MHz, RTS is transmitted at 3 Mbps, whereas for $B_{gu}<5$ MHz, RTS is transmitted at $R(B_{gu})$. As increasing $B_{gu}$ beyond 5 MHz does not further reduce RTS slot occupancy, both simulation and analytical results show that $\tau_{gu}$ converges. In contrast, since a larger $B_{gu}$ shortens the data transmission duration, $\lambda_{gu}$ continues to decrease even when $B_{gu}>5$ MHz. In addition, taking $\tau_{g}$ and $\lambda_{g}$ as representative examples, we further evaluate the impact of $\left[\delta_{g},R_{s}^{g},P_{eg}\right]$, with results shown in Figure 7a, Figure 7b, and Figure 7c, respectively. The results indicate that variations in the carrier sensing range $R_{s}^{g}$ have a more pronounced influence on $\lambda_{g}$ than changes in either the node density $\delta_{g}$ or the $P_{eg}$.

#### 6.1.2. Transmission Efficiency

**Figure 8:**
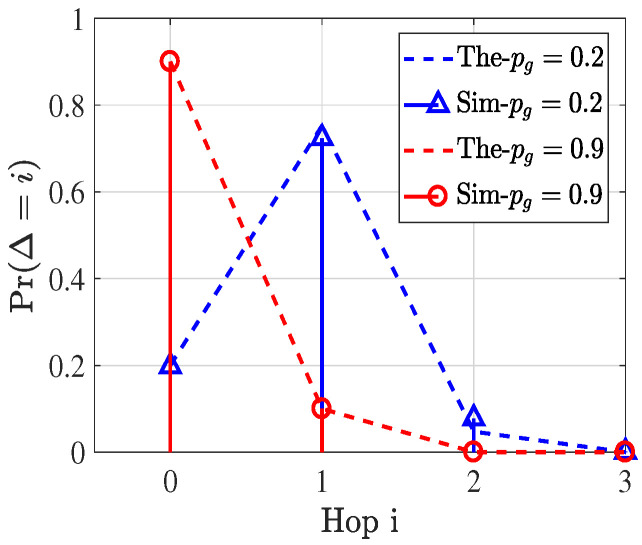
The probability that a node requires *i* hops to reach the nearest gateway node.

To validate the transmission efficiency in Equation (19), we examine the components ${\bar{h}}_{g}$, ${\bar{h}}_{u}$, and $p_{u}$ individually. Since ${\bar{h}}_{g}$ depends on the ground routing policy and is expressed in a general form, it is not explicitly validated in this subsection. For ${\bar{h}}_{u}$, we investigate the impact of the gateway ratio $p_{g}$ on the number of hops Δ required for a packet to reach its nearest gateway. In the simulation, packets are forwarded hop by hop toward the gateway with the minimum Euclidean distance, following a random routing strategy constrained by an angular limit of θ=π/2. Two representative cases, namely $p_{g}=0.2$ and $p_{g}=0.9$, are considered, with the corresponding results illustrated in Figure 8. As this scenario corresponds to a standard PPP void probability problem, the simulated results exhibit close agreement with the analytical model. Regarding $p_{u}$, since it is directly determined by the service capacity of the UAV, its validation is deferred to the evaluation of the E2E transmission success probability in the subsequent subsection.

#### 6.1.3. E2E Successful Transmission Probability

**Figure 9:**
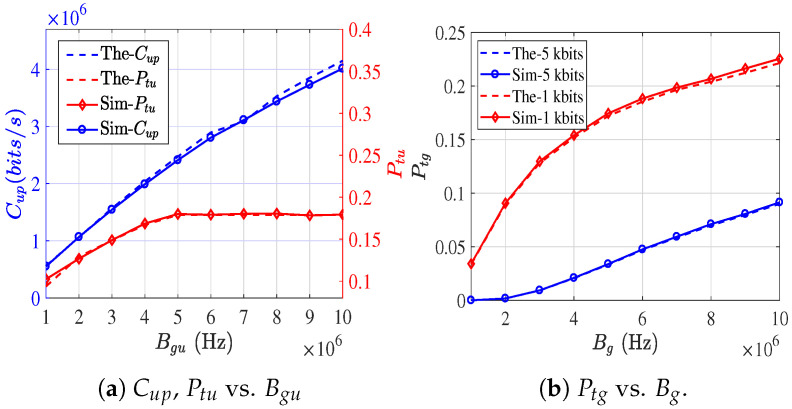
The impact of resource allocation on P2P transmission success probability.

For the E2E successful transmission probability, analytical models have been established for $P_{mu}$, $C_{up}$, $P_{mg}$, and $p_{u}$. The simulated and theoretical results illustrating the impact of $B_{gu}$ on $P_{mu}$ and $C_{up}$ are presented in Figure 9a. Consistent with the observations in the previous subsection, since the number of time slots occupied by RTS remains constant when $B_{gu}>5$ MHz, $P_{mu}$ converges to a stable value. In contrast, increasing $B_{gu}$ reduces the data transmission duration $T_{s}$, thereby leading to an increase in $C_{up}$. As shown in Figure 9b, $B_{g}$ has a pronounced impact on $P_{mg}$. It is worth noting that under the current simulation setting with a MAC data rate of 6 Mbps, a payload size of 5000 bits per packet is relatively large, resulting in a low success probability for G2G transmissions. To further validate the established $P_{mg}$ model, additional simulations were conducted with a reduced payload size of 1000 bits, and the corresponding results remain in good agreement with the analytical predictions.

At this stage, all models have been validated, and we now proceed to address the resource allocation optimization problem using these models.

### 6.2. Soft-Centralized Spectrum Resource Management

Based on the analytical expressions derived for $\lambda_{act}$, $\bar{E}$, and $\bar{P}$, we compute the AMIE metric and subsequently adopt it as the objective function of the MSF-PSO optimization framework. The decision variables $\left[c_{g},c_{ug},c_{gu}\right]$ are restricted to integer values within the range [0,20], while the coupling constraints in Equation (25) further limit the admissible combinations of $c_{ug}$ and $c_{gu}$. As a result, the feasible solution space remains relatively compact.

**Figure 10:**
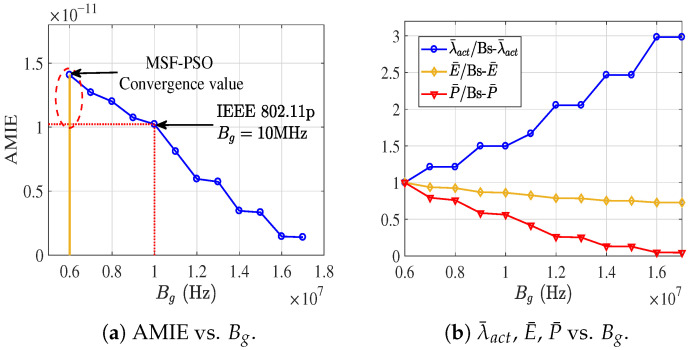
The impact of resource allocation on AMIE.

To provide a fair and transparent performance benchmark, we consider the conventional IEEE 802.11p fixed SCH allocation as a baseline scheme. In this baseline configuration, terrestrial G2G transmissions occupy one SCH, while G2U and U2G transmissions share another SCH, leading to a fixed bandwidth allocation of $B_{g}=B_{ug}+B_{gu}=10\;\mathrm{MHz}$. This setting has been widely adopted in existing UAMANET and UAVANET studies and therefore serves as a representative reference point. To verify the feasibility of the MSF-PSO algorithm and to demonstrate the necessity of resource optimization in UAMANETs, we first compute the AMIE curves as a function of $B_{g}$ via an exhaustive search over the entire feasible solution space. The resulting curves are shown in Figure 10a. Compared with the IEEE 802.11p fixed SCH allocation baseline, the proposed resource-domain partitioning strategy achieves a substantial performance gain. Under the considered parameter settings, allocating $B_{g}=6\;\mathrm{MHz}$ improves AMIE by approximately 40%, and the MSF-PSO algorithm consistently converges to the global optimum identified by exhaustive search.

Furthermore, Figure 10b presents the corresponding variations of the activated link density $\lambda_{act}$, the average transmission efficiency $\bar{E}$, and the E2E transmission success probability $\bar{P}$ with respect to $B_{g}$, thereby offering deeper insight into the underlying performance trade-offs relative to the baseline scheme. Since ${\bar{\lambda }}_{act}$, $\bar{E}$, and $\bar{P}$ differ considerably in scale, normalization is applied for visualization purposes. Specifically, the values at $B_{g}=6\;\mathrm{MHz}$ are used as reference points, and the normalized metrics $\frac{{\bar{\lambda }}_{act}}{\mathrm{Bs}\text{-}{\bar{\lambda }}_{act}}$, $\frac{\bar{E}}{\mathrm{Bs}\text{-}\bar{E}}$, and $\frac{\bar{P}}{\mathrm{Bs}\text{-}\bar{P}}$ are depicted in Figure 10b. Although increasing $B_{g}$ initially results in a higher $\lambda_{act}$, the overall AMIE converges at $B_{g}=6\;\mathrm{MHz}$. This phenomenon does not imply a degradation in multi-hop routing capability. Instead, it reflects a reliability-driven adaptation of the transmission strategy. Specifically, E2E communication over G2G links typically requires a larger number of hops and is subject to an exponential decay in success probability, namely ${\bar{P}}_{e2e}∼P_{p2p}^{H}$. When the per-hop reliability is limited, excessive G2G forwarding leads to frequent retransmissions and inefficient utilization of terrestrial spectrum resources. Under such conditions, the proposed framework naturally favors direct G2U transmissions via the UAV gateway, which offer shorter E2E paths and higher link reliability. Consequently, terrestrial bandwidth is primarily used as an efficient forwarding mechanism toward the gateway, rather than enforcing deep multi-hop routing irrespective of channel conditions.

It is important to emphasize that the proposed framework does not unconditionally prioritize either multi-hop G2G routing or direct G2U transmission. Instead, it evaluates the trade-off between hop count reduction and per-hop transmission reliability. As shown in subsequent figures, when G2G links exhibit sufficiently high reliability, multi-hop routing becomes advantageous and leads to substantial performance gains. Conversely, under unfavorable G2G channel conditions, the framework converges to direct gateway-assisted transmission as the optimal operating point.

**Figure 11:**
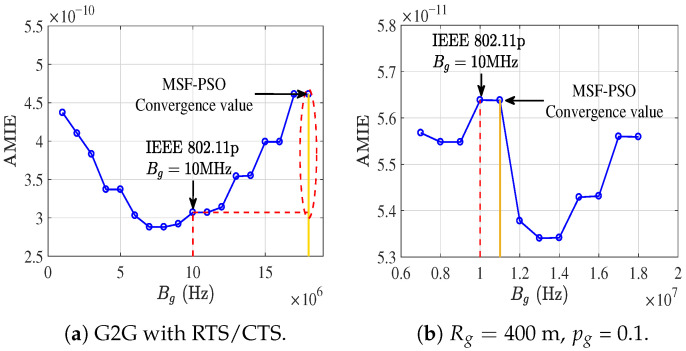
The relationship between AMIE and $B_{g}$ under different parameters.

Moreover, as discussed earlier, the effectiveness of spectrum resource allocation varies significantly across different network environments. When G2G transmissions additionally adopt the RTS/CTS mechanism, the resulting AMIE performance is presented in Figure 11a. Furthermore, under the parameter configuration $\left[R_{g},R_{s}^{g},p_{eg},p_{g}\right]=\left[400\;m,800\;m,0.5,0.1\right]$, the corresponding AMIE curve is illustrated in Figure 11b. Under these heterogeneous configurations, the AMIE curves exhibit diverse structural characteristics, including concave, convex, and monotonic behaviors. This observation indicates that the optimal spectrum resource allocation is highly sensitive to network parameters and channel conditions, thereby rendering analytical optimization intractable in general cases and strongly motivating the adoption of heuristic algorithms for efficient and robust optimization.

## 7. Conclusions

This paper studied a representative UAMANET in which UAVs form stable task clusters with ground nodes and serve as nodes of an airborne backbone. Using the AMIE metric, we quantified the effects of multi-hop routing, medium access contention, and transmission reliability. Our analysis shows that resource allocation introduces trade-offs among link activation density, transmission interruption probability, and end-to-end hop count and that CSMA/CA with RTS/CTS significantly improves G2U transmission reliability. To support efficient operation in decentralized environments, we proposed a soft-centralized resource management framework and jointly optimized resource allocation and access parameters using a MSFPSO algorithm. The results provide practical insights into designing UAMANETs with enhanced capacity, reliability, and scalability under heterogeneous network conditions. 

## Figures and Tables

**Table 1:** List of important notations used in this paper.

Notations	Descriptions
$N_{u}$	Number of UAVs
$N_{g}$	Number of ground nodes within the task cluster
β	SNIR for P2P transmission
α,F	Channel attenuation coefficient
A	Aggregate multi-hop information efficiency
$\lambda_{act}$	Density of activated links
$\bar{E}$	Average transmission efficiency
$\bar{P}$	Average E2E transmission collision-free probability
τ	Node’s transmission probability per idle time slot
$\bar{\varepsilon }$	Node’s actived probability per time slot
$R_{I}$	Interference radius generated by P2P transmission
$R_{s}$	The carrier sensing range

**Table 2:** Simulation parameters.

Parameter	Value
Area size	$\pi \;{\mathrm{km}}^{2}$
$\delta_{g}$	$2\times 10^{-5}\;\mathrm{nodes}/m^{2}$
$P_{eg}$, $P_{eu}$	0.8, 0.9
$W_{0},\;m,\;\beta$	16, 5, 7 dB
$R_{ug}$	≈1000 m
$R_{g}$, $R_{s}^{g}$	≈300 m, 600 m
Gateway node ratio	0.75
MAC Data rate when B = 10 MHz	6 Mbps
Payload size	5000 bits
Total bandwidth	20 MHz
Minimum bandwidth allocation unit	1 MHz
Routing model	Random routing
MSF-PSO Population size	5
MSF-PSO Max iterations	10
MSF-PSO Inertia weight *w*	0.7
MSF-PSO Cognitive coefficient $c_{1}$	1.5
MSF-PSO Social coefficient $c_{2}$	1.5
MSF-PSO Position bounds	[0, 20]

## Data Availability

The data presented in this study are available on request from the corresponding author.
